# Mobile DNA: an evolving field

**DOI:** 10.1186/1759-8753-5-16

**Published:** 2014-06-05

**Authors:** Marlene Belfort, Luciano Marraffini, Todd Macfarlan, Jef Boeke, Keith Slotkin, Harmit Malik, Lynne Maquat

**Affiliations:** 1 Department of Biological Sciences, Biology Building, Room 126, University at Albany, State University of New York, 1400 Washington Avenue, Albany, NY 12222, USA; 2 Laboratory of Bacteriology, The Rockefeller University, 1230 York Avenue, New York, NY 10065, USA; 3 Unit on Mammalian Epigenome Reprogramming, Building 6B, Room 2B211, 6 Center Drive, Bethesda, MD 20892, USA; 4 Institute for Systems Genetics, NYU Langone University School of Medicine, 550 First Avenue MSB238, New York, NY 10016, USA; 5 570 Aronoff Laboratory, Ohio State University, 318 West 12th Avenue, Columbus, OH 43210, USA; 6 Division of Basic Sciences, Fred Hutchinson Cancer Research Center, P.O. Box 19024 Seattle, WA 98109-1024, USA; 7 University of Rochester Medical Center, School of Medicine and Dentistry, 601 Elmwood Ave, Box 712, Rochester, NY 14642, USA

## Abstract

During the Mobile Genetic Elements and Genome Evolution Keystone Symposium in March 2014, the Editors of *Mobile DNA* caught up with a panel of conference speakers to select key advances in the field, and hear their thoughts on where mobile DNA research is going.

## Introduction

At a time when transposable elements are gaining increased interest as powerful engines of genome evolution, the Keystone Symposium ‘Mobile Genetic Elements and Genome Evolution’ in March 2014 aimed to explore the cutting edge of mobile DNA research. In particular, the conference focused on recent innovations in the study of transposon biology and genome evolution, and the evolutionary arms race that exists between mobile DNA and their hosts. In addition, findings around active transposition during neurogenesis and in tumor cells, and its potential role in brain development and cancer, was a key topic of discussion.

With so many ongoing questions and controversy within the transposable elements community, the Editors of *Mobile DNA* spoke with a panel of speakers at the meeting to hear their thoughts on key advances in the field, the big questions at the moment and where research is going.

The panel represent divergent specialties involving mobile genetic elements, a field which is increasingly interacting with other fields as the significance of transposable elements is further understood.

## What’s the most exciting advance recently?

### Marlene Belfort

I am really excited because the very fundamental work that is done in mobile elements is becoming so hugely applied. For instance, Haig Kazazian’s work [1] on the role of LINE elements in various diseases, the fact that LINE elements have different patterns in cancer, and not knowing whether that’s causal or consequential. Then, at the other end of the spectrum, the use of transposons as landmarks in the genome to do high-throughput sequencing and further advance the field. Of course, part of the advance is because of our ability to sequence these genomes so we can really follow elements and know where they’re going. Now it’s come full circle that the very elements that we can follow are becoming useful in generating the next generation of data.

### Luciano Marraffini

I think one really interesting development that relates to mobile genetic elements is the finding of CRISPRs in phages. There’s a paper by Andrew Camilli and coworkers in *Nature*[2], where they found that phages carry CRISPRs. The authors isolated and sequenced *Pseudomonas aeruginosa* phages and found that some contained CRISPR-Cas systems. Through an elegant set of experiments they found that the CRISPRs on the phage were used to attack an antiviral chromosomal island of a bacterial host. So the phages hijacked CRISPRs for their own benefit. I think that’s a very interesting twist in terms of mobile genetic elements, that now phages can use CRISPRs too.

### Todd Macfarlan

My lab studies retroviral elements that are reactivated in mouse tissues, and now there’s been a number of reports showing that human endogenous retrovirus elements are also expressed. HERVs appear to serve as promoters for important long non-coding RNAs, and are probably involved in rewiring gene regulatory networks in human development. So it’s satisfying to hear that distinct retroviruses have been co-opted for regulating early embryonic gene expression in mammals (an elegant case of convergent evolution). We shouldn’t just be thinking of viruses as sequences that are potentially harmful or damaging; mammals have decided to use viruses to our selective advantage.

## What are the big questions at the moment?

### Harmit Malik

For me personally, the big questions haven’t changed. What are the evolutionary strategies that the elements take to thwart the host defences that are in place? What kind of arms race is played out between mobile elements and these host defences? And can we use this new found information, of the scale of information, to actually discover more important host defences that are going to be important for not just the evolutionary questions that I’m interested in, but also biomedical questions where mobile elements may have very important roles in many diseases.

### Jef Boeke

Some of the questions about cause or consequence in somatic transposition: what’s really the extent of it, and what’s the biological significance of it at the end of the day? I confess I’m still a sceptic on some aspects of the findings and the way that people interpret them.

As one example, I think the work from Gage and Moran and Faulkner [3-6] and others has clearly demonstrated that there’s hopping going on in neurons and we’re beginning to get some sense of what the numbers are. But I’m fundamentally a selfish DNA guy, and the idea that these elements would be jumping around to provide a function is still very difficult to swallow. I think an alternative explanation for those findings, that needs to be considered, is that in the brain and in the germ line many, many genes are expressed. It’s a very permissive environment for expression. So perhaps this is a common feature of the transcriptional environment between these two tissues – they’re both permissive for transposition. Perhaps some of the same transcription factors are used? This might explain why we see increased transposition in these very selected environments.

### Luciano Marraffini

In CRISPR, there’s no doubt that the biggest question is their spacer acquisition; i.e. the process by which a short sequence of the phage DNA is integrated into the CRISPR locus. I think this is one of the most interesting parts of CRISPR immunity: the ability of an organism to adapt directly using information from their environment. It’s not precisely Darwinian, it’s more like Lamarckian evolution. But we really don’t know anything about how it works, and until then we may need to wait to call it a Lamarckian process.

A second question is the tremendous diversity of CRISPR-Cas9 systems. For many of them, we don’t know what they do. Perhaps they are serving other functions different than anti-phage defence, and that would be very interesting. But if not, it would also be very interesting to understand why is there so much diversity among CRISPR systems.

### Lynne Maquat

There is the quote “Nothing in biology makes sense except in the light of evolution” [7]. If we look at where we are now, evolutionarily, and try to *de novo* figure out how we got here, we will never be able to recapitulate the ordered development of, as one example of particular interest to me, our impossibly complex post-transcriptional regulatory network. This is because processes were constantly being built upon existing processes. Considering that the bulk of our genome is transcribed, and a lot of the resulting RNAs are regulatory, our task at hand now is to figure out the functional interplay of these RNAs with each other and with other molecules in the cell. I’m sure that some of these transcripts have no function, but a surprisingly large number probably do. They don’t encode protein, so what are they doing in the nucleus, in the cytoplasm or in both?

### Todd Macfarlan

I think there is a lack of strong evidence for a functional requirement of most transposable element sequences. There is growing circumstantial evidence (in some cases quite weak) that if you knock-down a given transposable element using siRNA approaches, there are subtle phenotypes. With the advent of new genome editing tools, I expect there will be much stronger genetic evidence of a requirement for transposable element-mediated regulation of gene expression. Nonetheless there has been a report showing that even removal of a single retroviral LTR (within the DICER gene) can lead to a lethal phenotype (in the mouse) by preventing the expression of an alternative splice variant of DICER. To me, this is the best proof yet for a requirement for retrovirus regulatory activity.

### Keith Slotkin

In my field, which is more the epigenetics field, a lot of us ask the same biological question: While we understand how a transposable element can be maintained in a silent state and repressed over time, we don’t understand its original recognition and its initial triggering to be silenced. That’s a process and molecular mechanism that’s been very tough to get at and a lot of labs are now going after this question quite aggressively.

## What directions can you see the field going in?

### Jef Boeke

One of the areas that I’m personally very excited about, and a road that I’ve decided to go down, is to build genomes from scratch where every last nucleotide of mobile DNA has been ruthlessly exterminated by synthesis. That’s one of the goals of our synthetic yeast genome project. We’re doing the experiment that we’ve talked about at the bar at every transposon meeting, which is taking out all the transposon DNA from an organism and asking what will happen. Now, I’m betting that we will get away with it in yeast and we won’t see any phenotype that we can attribute to that. I would not be so sanguine about saying that about humans or mammals; I think we would definitely see phenotype differences.

### Marlene Belfort

I think that the field will continue to not only provide insight into genome dynamics in healthy populations of all different kinds, but in disease populations. An understudied area is the responsiveness of mobile elements to stresses of various kinds. So I think part of the distribution of these elements results from radiations of the elements during times of stress.

I see the use of transposable elements in various forms of genotyping in medical applications. I anticipate both exploitation of the elements for different practical purposes and, again, more fundamental insight – they’re all in our future. But thinking about key directions the field is going in, the thing that really popped into my mind is: we know that they’re responsive to stress but we have just scratched the surface. That’s where evolution lies – our responsiveness to stress and our adaptations to the stress – and I think that the various kinds of mobile elements including introns play a huge role here.

### Keith Slotkin

I think a few years ago it was clear that the field had not taken to the rapid explosion of sequencing technologies and other new genome-wide assays. There are a lot of new tools available, but this community had been a little bit slow to catch up with the new technology. Now it’s evident that these new tools have caught on and people have adapted and adjusted their research plans. The number of people doing deep sequencing and looking at transposable elements using large genome-wide datasets that they’ve produced has increased at an exceptional rate.

People in our field are now using state of the art technology, such as single-cell neuron sequencing. That’s a good use of new technology, and over the next five or ten years there’s going to be even a larger push for even newer technology in this field such as long-read deep sequencing, which will directly benefit research on repetitive elements such as transposable elements.

### Lynne Maquat

I got into this field through studying the molecular basis of human diseases. I think that with a better understanding of how gene expression occurs and how it’s regulated, we’re going to have a much better idea of how to develop effective therapies. It’s clear that RNA can be used as a therapeutic target and RNA can also be used as a therapeutic tool. With the new information we are getting, including information on how RNA-binding proteins influence RNA metabolism, I think we are entering a new phase of developing therapeutics. That’s very exciting and something that we and many others are pursuing.

### Harmit Malik

I think the field is at a little bit of an inflection point; I feel that technology is both a blessing and a curse in some ways. The blessing is that we can ask questions that we never really had the ability to ask. On the other hand, the risk of these new methods is that you get lazy in terms of the types of questions you ask, and you see a lot of the “me too” adjective, where a lot of people are doing very similar things to profile mobile elements and different things. Now, that’s technologically challenging at the present moment; but my concern is that once we’ve answered questions, we don’t want to lose sight of the very basic intellectual questions that still remain to be addressed.

## Competing interests

The authors have no competing financial interests to declare.
